# Permanent Retention of Antimony in the Living Organs

**Published:** 1848-03

**Authors:** John M. B. Harden

**Affiliations:** Correspondent Acad. Nat. Sci., Philadelphia


*Permanent Retention of Antimony in the living Organs.*—(Presented to the Academy of Sciences on the 22d June, 1846.) Translated from the February No. of the Annales de Chemie et de Physique. By John M. B. Harden, M. D., Correspondent Acad. Nat. Sci., Philadelphia.—In studying the passage of Antimony through the system when given in the form of an emetic, M. Laveran and myself have had occasion to note its permanent retention in the different organs, and its gradual elimination after a long time. This observation leading us, as it did, to attribute to the economy the faculty of retaining certain principles foreign to the composition of the organs a longer time than had before been suspected, made it necessary to follow it up and ascertain more particularly the precise length of time to which it could be extended. Legal medicine is greatly interested in this subject—and Pathology no less so. We have every day reports of cases of Poisoning of protracted duration—with equal care should we chronicle the facts which tend to demonstrate that a very minute quantity of any metallic substance, when once introduced into the tissues remains permanently there, and may be transmitted, as we shall see, from the female to its young.

In order to make correct observations upon this subject, the ordinary cases occurring in disease are not sufficient. We have found it necessary, therefore, to administer the emetic to dogs. For this purpose six dogs were kept up and so fed as to support a long confinement. To the daily food of each dog were added a few grains of tartar emetic.

The first trials with the emetic enabled us to determine, first, the comparative value of the methods proposed for detecting the antimony in the tissues; secondly, to establish the fact of its introduction into the different organs of the body; and thirdly, to prove its retention in these organs during a certain limited period of time, to determine which, however, a new experiment was necessary, in which six other dogs were kept up for many months.

In those dogs which died during the first week of the experiment the antimony was found in the liver, the heart, the muscles, the coats of the intestines, and the lungs. The brain, the bones and the fat, were entirely free from any traces of it. At the end of fifteen, twenty, and twenty-five days, the amount of the metal in each organ was found to be the same, its quantity not being sensibly diminished. We must add, that in these early cases of poisoning from antimony, the proportion absorbed by the liver was comparatively enormous. 600 grammes of different tissues in which anatomy was found, furnished scarcely as many spots as 100 grammes of the liver.

Before entering into a detail of these new experiments, however, which appear to me sufficiently interesting to be given in full, I will describe the method which I have employed for the purpose of detecting the antimony in the tissues. This method consists in the employment of hydrochloric acid and the chlorate of potash. By causing them to act successively upon the organic matters they are almost entirely destroyed. The antimony is precipitated upon a very thin lamina of tin. The two metals are then dissolved, and we introduce the solution into Marsh's apparatus.

But to be more particular. We weigh 50 to 100 grammes of the tissue to be examined, for example, the liver, the intestines or muscular flesh: it is cut in pieces, while fresh, and introduced into a glass vessel containing one litre, and upon this we pour pure fuming hydrochloric acid to an amount equal in weight to one half of the organic matter. The mixture is then placed upon a sand-bath, not hot enough, however to cause the acid to boil. After digesting in this way five or six hours, it is then made to boil, and immediately we add to the mixture small bits of the chlorate of potash. Fifteen or sixteen grammes of the chlorate is added to every 100 grammes of the organic matters. This addition should be made while agitating the mixture and should consume about fifteen minutes.

As soon as the chlorate has been introduced, the fluid should be filtered while boiling hot. Upon the filter will be found a yellowish or brown matter, resenoid, insoluble, but varying according to the nature of the tissues. The filter with the insoluble matter is now to be washed in a little distilled water, and into the filtered fluid, which is limpid and often colorless, we place a thin lamina of tin. If the antimony is abundant, the tin will be strongly blackened; if not, it will be barely tarnished, and will be covered over by some black spots. However, it may be, after keeping it in the fluid for twenty-four hours, we remove the tin and introduce it into a small flask, and pour upon it a quantity of hydrochloric acid sufficient to dissolve it in the cold after a few hours. Should a few blackened portions remain undissolved, we decant, and by the aid of a few drops of nitric acid the solution is effected, and we mix them afterwards with the dissolved mass.

The quantity of tin dissolved should not be too large, but we may reduce as much of it as we wish by successive precipitations upon lamina of tin having a very small surface.

I pass now to a consideration of the experiments upon the six dogs which were kept up for many months after the use of the antimony.

On the 8th day of January, these animals were put upon a regimen consisting of bread and pieces of meat. Their rations were mixed every day with a solution of tartar emetic. Each dog ought to have received, according to the common ratio, about 4 decigrammes of the tartarized antimony for his part each day. At the commencement of the experiment the dogs evinced a very strong appetite; which seemed to be increased by the use of the new condiment; but in a short time we witnessed very opposite results. Their voracity was replaced by a marked disgust, and on the 24th of January two of the dogs refused to eat. The proportion of antimony was now diminished to one half, but on the 28th the use of it was given up, the dogs did not touch it again in their food, and yet all of them showed a very marked leanness. Thus the antimonial regimen, which lasted ten days, furnished to each day about three grammes of the emetic.

After having given up the use of the antimonial, and returned to ordinary food, four of the dogs regained their appetites, and by degrees their former healthy condition; but there were two which could not overcome the effects of the metallic poison. They continued to exhibit a complete inappetency, were reduced to the last degree of emaciation, and on the third of February one of them died. Its body was extremely emaciated; the organs generally did not exhibit any great alteration except the liver, which was friable, and remarkable for its volume. It was weighed and compared with the total weight of the body we found the proportion to be as one to 12—(1:12.)

As this ratio appears to me very interesting and worthy of note, I will state at once, that in the cases of dogs in a good state of health, we have found the following proportions between the liver and the mass of the body.

1:32
1:40
1:24

In regard to the distribution of antimony into the tissues of this first dog, it was general, without any apparent preference, for one or another organ: the liver, the muscular flesh, the coats of the intestines, the lungs, the brain—all seemed to be equally filled with it. The animal seemed to die from a sort of antimonial diathesis.

The second dog lived a few days longer, but died on the 10th February. During the few last days, he was affected with a continual nervous tremor; the hinder legs were also affected in a singular manner, they would all of a sudden give way, and the progress of the animal would be instantly arrested. Yet the organs did not present any striking alteration. The liver was friable, as in the preceding case, and voluminous, being in the ratio of the mass of the body of 1:10. The antimony was in this case also found in every part, but the brain seemed to have retained rather more than any other organ.

Our observations were now confined to the four surviving dogs. At the end of twenty days they were all restored, their appetite and natural vivacity having returned. One of them got away and was not again examined; another died suddenly. A post mortem examination showed that his death was occasioned by the passage of a lumbricus through the coats of the intestine into the peritoneal cavity in which it was found. A search for antimony, in this case, showed a very peculiar distribution of the metal. It was found in a very notable proportion in the liver and in the fat, but it was particularly accumulated in the BONES. It is necessary to state here, that this dog died of a *true accident* six weeks after having taken the antimonial, and that he had improved already a great deal in flesh. The antimony of course had lodged in organs where its retention was consistent with the regular exercise of all the functions. This remark acquires a peculiar value when we take into account the very similar distribution in the last two dogs which were killed, one after three and a half and the other after four months. I must declare also that the metal was in *these*, in as large proportions as in those which died during the very earlest periods of our experiments.

In the dog which was killed three months and a half after, having ceased in the use of the antimonial, the metal was found in the largest quantity in the fat. The liver contained some of it as well as the bones and the other tissues; but 50 grammes of the fat furnished as much as 500 grammes of all the other tissues put together. The ratio of the liver to the general mass had been restored to the normal state of 1:27.

In the dog which for four whole months had taken none of the antimony the metal was found accumulated in the bones; the liver contained also a large quantity of it: all the other tissues gave very little traces of it. One hundred grammes of the bones yielded a quantity of the metal sufficient to cover over with perfect metallic spots the surfaces of three porcelain cups with a diameter of ten centimetres. The liver had attained a relative volume of 1:24.

I will conclude these observations, which so strongly attest the permanent retention of antimony in the living tissues, by mentioning the case of a young bitch to which the emetic was given during five days, and about fifteen days before the birth of her young ones. The dog and her young were killed immediately after birth, and the livers of the puppies were found to contain a notable quantity of antimony.

It is not an easy matter to know what conclusion should be deduced from the preceding experiments. Some of them introduce new elements into certain questions connected with medical jurisprudence, the value of which will be at once perceived; others belong rather to the domain of pathology.

Although the antimony seems to become organized, we cannot yet affirm that it is ever fixed in our tissues: neither must we suppose, in advance, that the facts revealed by the administration of antimony will also hold true with other metallic poisons. We must wait on experiment. So to affirm that a metal detected must have come from a recent ingestion, or to fix its origin and the moment of its introduction into the economy, it is necessary to wait: we must address ourselves to the task and vary our experiments.

In regard to the distribution of antimony in the organs, I have been struck with its relation to the physiological effects which have been mentioned above. Has the antimony penetrated simultaneously all the important organs, such as the lungs, the brain, and the coats of the intestines, the animal yields to the general poisoning, and seems to die all over at the same time from the effects of an extreme emaciation. Has the metal been lodged in the brain? we find the same general disease, but death takes place under an array of nervous symptoms which indicate the principal seat of the poison.

Let the metal, on the other hand, be deposited in organs of less sensibility and less general sympathy; such as the cellular and osseous systems, and the effects of the poison will soon be effaced, so as to lead us to suppose that it was entirely eliminated.

From this novel development in relation to poisoning by antimony, we are naturally led to suspect similar conditions in cases of poisoning with lead. May it not be by a kind of special localization that some privileged constitutions escape the poisonous effects of the last metal? and does not the concentration of morbid symptoms upon the abdomen, upon the nervous system, or upon the limbs, show that the lead occupies corresponding regions?

This is one of the numerous methods by which may be explained all those affections in which the presence of noxious principles, foreign to the economy while in the normal condition, is for the present suspected rather than demonstrated.

The enormous development of the liver, in consequence of the administration of the antimony, is also a fact which must not be passed over unnoticed. The percussion of the organs is at present practised with so much skill that we shall not be long in ascertaining whether the use of the antimony in man is attended likewise with the rapid enlargement.

*Southern Medical Journal.*

